# Dry Sliding Wear Behavior and Mild–Severe Wear Transition of Mg97Zn1Y2 Alloy at Elevated Temperatures

**DOI:** 10.3390/ma11091735

**Published:** 2018-09-14

**Authors:** Liang Li, Jihe Feng, Ce Liang, Jian An

**Affiliations:** 1 Key Laboratory of Automobile Materials, Ministry of Education, Jilin University, Changchun 130025, China; liangliang-2005@163.com; 2 Department of Materials Science and Engineering, Jilin University, Changchun 130025, China; 3 Faw Jiefang Automobile Company Limited, Changchun 130011, China; 18204312080@163.com

**Keywords:** sliding wear, non-ferrous metals, hardness, wear testing, surface analysis, high temperature

## Abstract

Dry sliding wear behavior of Mg97Zn1Y2 alloy was investigated at test temperatures of 50–200 °C under three sliding speeds of 0.8 m/s, 3.0 m/s and 4.0 m/s. The wear mechanisms in mild and severe wear regimes were identified by examination of morphologies and compositions of worn surfaces using scanning electron microscope (SEM) and energy dispersive X-ray spectrometer (EDS), and from which wear transition maps under different sliding speeds were constructed on rectangular coordinate systems with applied load versus test temperature axes. It is found that under each sliding speed condition, mild–severe transition load decreases almost linearly within the test temperature range of 50 °C to 200 °C. Microstructure observation and hardness measurement in subsurfaces identify that the softening effect generating form dynamic crystallization (DRX) is the dominant mechanism for the mild–severe wear transition at elevated temperatures. The mild–severe wear transition at 50–200 °C follows the contact surface DRX temperature criterion, and the transition loads can be well evaluated using the criterion.

## 1. Introduction

Mg97Zn1Y2 alloy has attracted much attention since a type of high-strength Mg97Zn1Y2 alloy was first prepared by rapidly solidified powder metallurgy (RS/PM) technique in 2011 [1,2,3,4]. RS/PM Mg97Zn1Y2 alloy exhibits super high yield strength above 600 MPa and a decent elongation of 5% at room temperature, and yield strength of 510 MPa at 150 °C, which are much superior to the room and elevated-temperature mechanical properties for most of commercial magnesium alloys. Furthermore, it is found that the long-period stacking ordered (LPSO) phase X-Mg_12_ZnY in the matrix of Mg97Zn1Y2 alloy contributes greatly to high-temperature strength, ductility and thermal stability through fiber-strengthening effect and kink bands and so on [5,6,7]. Therefore, Mg97Zn1Y2 alloy is expected to have great potential as a metallic material or a matrix of magnesium alloy composites in tribological applications where aluminum alloys have been traditionally used, such as plastic mold, low-load bearing gear, clutch piston, sliding bearing. Mg97Zn1Y2 alloy also showed a better wear resistance at room temperature as compared with the most widely used AZ91 magnesium alloy [8]. As for tribological applications of aluminum and magnesium alloys, the mild–severe wear transition is of great interest to researchers and engineers, since mild wear regime is usually regarded as a safe wear region. In a mild wear regime, wear proceeds in such a steady state that the resulting wear rate is allowable, and surfaces are only damaged slightly [9,10]. Recently, based on a comprehensive investigation conducted on mild–severe wear transition of AZ91 alloy by Chen and Alpas [11], a contact surface dynamic crystallization (DRX) temperature criterion for mild–severe wear transition of magnesium alloys during the room-temperature sliding wear was established in our previous studies, and it has been successfully applied to several magnesium alloys such as AZ31, AZ51, AS31 and Mg97Zn1Y2 alloys [12,13,14,15]. The mild–severe wear transition loads were well predicted under different sliding speeds using DRX dynamics. Moreover, our findings confirmed that Mg97Zn1Y2 alloy demonstrated a better resistance to mild–severe wear transition than AZ alloys and AS31 alloy during room-temperature sliding wear [16].

It should be pointed out that the superior elevated-temperature mechanical properties make Mg97Zn1Y2 alloy have potential in elevated-temperature tribological applications. Even though there have been a few investigations into the elevated-temperature wear behavior of magnesium alloys including AZ91D, AZ91 + 3 wt.% RE alloys and Mg/SiC composites [17,18,19,20], the elevated-temperature wear mechanisms and mild–severe wear transition mechanism for magnesium alloys have not yet been well clarified, especially for those magnesium alloys with high thermal stability. So far the elevated-temperature wear behavior of Mg97Zn1Y2 alloy has not been investigated systematically, mainly because the wear process at elevated temperatures is very complicated. The elevated-temperature wear tests, unlike room-temperature ones, are considerably influenced by three experimental parameters, i.e., applied load, sliding speed and test temperature. Therefore, the mild wear regimes (safe wear regions) of Mg97Zn1Y2 alloy at elevated temperatures have rarely been determined, and there have not been sufficient elevated-temperature wear data to provide for engineering reference.

In the present contribution, the wear behavior of Mg97Zn1Y2 alloy was investigated within a temperature range of 50–200 °C under three sliding speeds conditions, i.e., 0.8 m/s, 3.0 m/s and 4.0 m/s. The influences of applied load and test temperature on wear mechanism were analyzed using SEM and EDS techniques to establish elevated-temperature wear transition maps. The mechanism for mild–severe wear transition at elevated temperatures was explored by analyzing the changes of microstructure and hardness in subsurfaces during sliding wear. The feasibility of applying contact surface DRX temperature criterion for mild–severe wear transition at test temperatures of 50–200 °C was assessed by comparing the measured transition loads with the calculated ones using the proposed criterion.

## 2. Experimental Details

The material tested was an as-cast Mg97Zn1Y2 (in atomic percentage) alloy, which was produced into an ingot of 95 mm in diameter and 200 mm in length through conventional casting route. The details can be seen in study [16]. Dry sliding wear tests were performed on a MG2000 high-temperature and high-speed tribometer (Chengxin Test Equipment Manufacturing, Zhangjiakou, China) in pin-on-disc contact mode. Pin specimens of 6 mm in diameter and 12 mm in height were machined from Mg97Zn1Y2 for wear testing. The counter disks of 70 mm in diameter and 10 mm in thickness were made of AISI 5150 steel quenched to hardness 57 HRC. AISI 5150 is middle carbon chromium steel that is commonly used for making crankshaft and piston pin. The track radius was 30 mm. The contact surface of pins and disks were polished to a roughness of about Ra = 0.4 μm before each wear test. The sliding speeds were 0.8 m/s, 3.0 m/s and 4.0 m/s, respectively. Under each sliding speed, wear tests were conducted by applying load from a low load 10 N or 20 N until 260 N or occurrence of surface melting at test temperatures of 50 °C, 100 °C, 150 °C and 200 °C, respectively. The elevated-temperature wear tests were performed in a resistance split furnace (Jujing Precision Instrument, Shanghai, China) with an accuracy of ±5 °C, and pin and disc were held at each desired wear temperature for 15 min to stabilize the environmental temperature before commence of the wear tests. The sliding distance maintained 565 m. The volumetric wear loss was determined by the pin length reduction, which was measured by a digital precision micrometer (Shoufeng Precision Instrument, Shanghai, China) with the accuracy of 0.001 mm. The wear rate under a given sliding condition was an average of at least three wear specimens. In order to ensure the accuracy of test results, the wear rate data adopted were below a relative average deviation of 5%.

The morphologies and chemical compositions of worn surfaces were examined with a TESCAN VEGA3 scanning electron microscope (TESCAN, Brno, Czech Republic) equipped with an energy dispersive X-ray spectrometer (Oxford Instrument Company, Oxford, UK). The cross-sectional microstructures in subsurfaces after wear tests were observed parallel to the sliding direction under a LEXT-OLS3000 confocal scanning laser microscope (Olympus Corporation, Tokyo, Japan). The variation of Vickers microhardness in subsurfaces with depth from surface was measured by a HVS-1000 microhardness tester (Weiyi Metallographic Test Instrument, Guangzhou, China). The applied load and dwell period were 0.49 N and 15 s, respectively.

## 3. Results and Discussion

### 3.1. Effects of Applied Load and Test Temperature on Wear Rates

**Figure 1:**
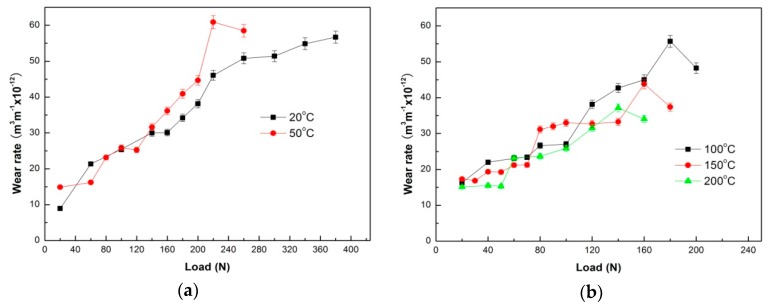

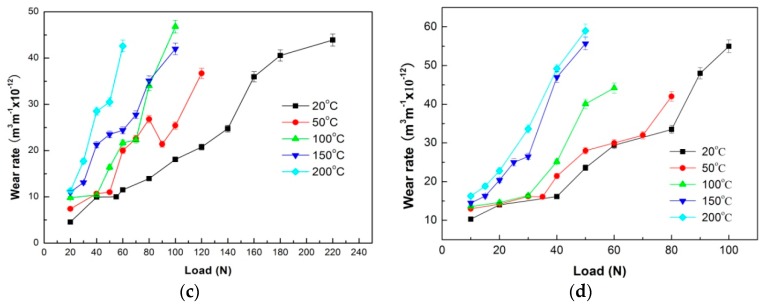
Variations of wear rates of Mg97Zn1Y2 alloy with applied load under different sliding conditions: (**a**) 20–50 °C, (**b**) 100–200 °C, 0.8 m/s, (**c**) 3.0 m/s, (**d**) 4.0 m/s.

The wear rates of the Mg97Zn1Y2 alloy obtained at test temperatures of 50–200 °C under conditions of three sliding speeds are plotted as a function of applied load in Figure 1a–d, respectively. For reference, the wear rate data obtained at 20 °C under the given sliding speeds are also included in Figure 1 [16]. Under the condition of intermediate sliding speed 0.8 m/s, at low test temperatures of 20 °C and 50 °C, the wear rates increased moderately with applied load increasing before a sudden rising at 160 N and 130 N respectively, and then went up with a large slope until a high level or occurrence of surface melting by naked eyes, as shown in Figure 1a. At the two transition loads, the wear rates were 25.3 × 10^–12^ and 30.1 × 10^−12^ m^3^m^−1^, which were close to those at mild–severe wear transition loads under the same sliding speed during room-temperature wear testing for conventional magnesium alloys such as AZ31, AZ51, AZ91 and Mg-3Al-0.4Si alloys [11,12,13,14]. Therefore, it can be considered that when test temperatures are 20 °C and 50 °C, wear behavior transits from the mild to the severe at 160 N and 130 N respectively. While the wear rates varied differently with applied load at high test temperatures of 100 °C, 150 °C and 200 °C, as shown in Figure 1b. At first the wear rates also increased gently with applied load increasing or even maintained a plateau regardless of applied load, for example, within 20–50 N at 200 °C, but then they rapidly rose when applied loads exceeded certain values, namely 100 N at 100 °C, 70 N at 150 °C and 50 N at 200 °C. It is found that the wear rate at the transition load decreases with increasing test temperature, ranging from 15.3 × 10^−12^ to 25.4 × 10^−12^ m^3^m^−1^. These loads also probably correspond to the mild–severe wear transition loads, if compared with the transition loads and corresponding wear rate range of 16.4 × 10^−12^–26.2 × 10^−12^ m^3^m^−1^ of Mg-3Al-0.4Si-0.1Zn alloy under the same sliding conditions [21]. Furthermore, it is noted that unlike the continuously increasing of wear rate at 100 °C after a sudden rising at load of 120 N, the wear rate curves at temperatures of 150 °C and 200 °C presented two distinguished plateau regions, which were within load ranges of 80–140 N and 60–80 N, respectively. The presence of plateaus is apparently contrary to popular expectation that wear rate would rapidly increase with increasing applied load in severe wear regime. The reason for formation of such a wear rate plateau after mild–severe wear transition will be explained later in Section 3.2, from the viewpoint of accumulation of Mg12ZnY phase in the surface layer material during sliding wear.

Under the conditions of high sliding speeds of 3.0 m/s and 4.0 m/s, the wear rate curves presented two distinct characteristics: firstly, there existed a clear transition point at a certain load depending on test temperature and sliding speed; secondly, the rising rate with applied load was much more pronounced with test temperature increasing, as shown in Figure 1c,d, respectively. For example, the transition loads were 55 N at 20 °C, 50 N at 50 °C, 40 N at 100 °C, 30 N at 150 °C and 20 N at 200 °C under the condition of 3.0 m/s, while they were 40 N at 20 °C, 35 N at 50 °C, 30 N at 100 °C, 15 N at 150 °C and 10 N at 200 °C under the condition of 4.0 m/s. The wear rates at these transition loads were apparently influenced by the sliding speed, varying from 10.0 × 10^−12^ to 13.1 × 10^−12^ m^3^m^−1^ at 3.0 m/s and 16.1 × 10^−12^ to 17.3 × 10^−12^ m^3^m^−1^ at 4.0 m/s, much lower than those at 0.8 m/s. Moreover, the test temperature has a much stronger effect on wear rate variation with applied load at high sliding speeds than at 0.8 m/s, since the slope of wear rate curve increases fast with increasing test temperature when applied load is larger than transition load. Wear rate curves at sliding speeds of 3.0 m/s and 4.0 m/s, unlike the ones at 0.8 m/s, do not tangle together after mild–severe wear transition.

### 3.2. Wear Mechanisms

The above-mentioned transition loads in the wear rate curves were confirmed as the mild–severe wear transition loads by subsequent SEM analysis of worn surfaces, since a typical wear mechanism happened in severe wear regime for magnesium alloys, i.e., severe plastic deformation was observed dominating the wear process when applied loads exceeded the transition loads. Under the given sliding conditions, wear mechanisms operating within mild and severe wear regimes were identified by SEM examination and EDS analyses of the worn surfaces.

**Figure 2:**
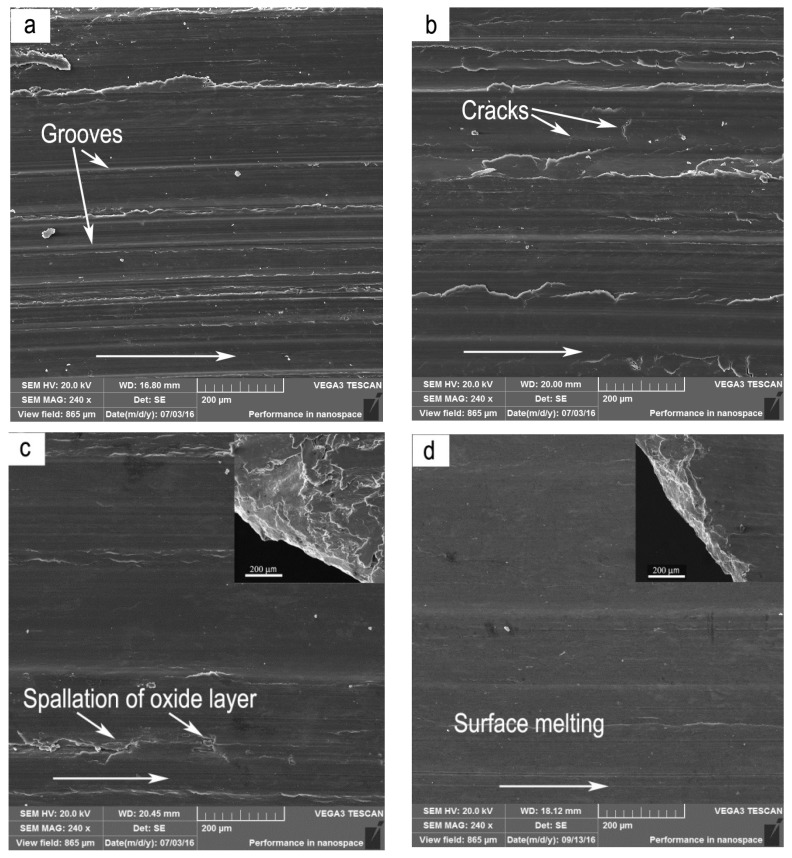

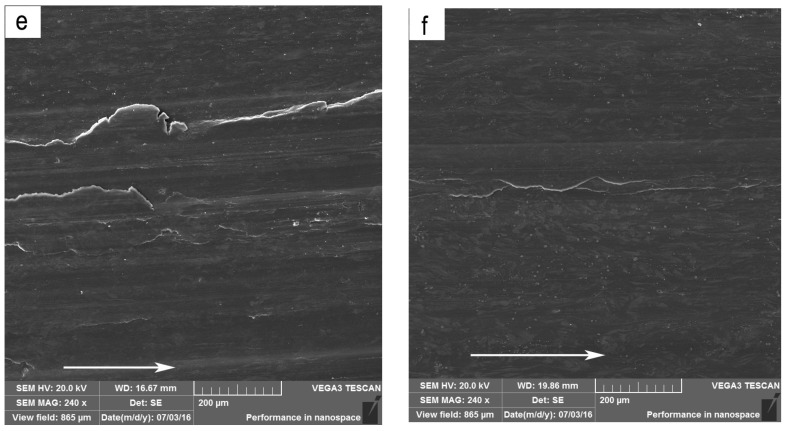
Worn surface morphologies at different applied loads and test temperature under 0.8 m/s: (**a**) 20 N, (**b**) 60 N, (**c**) 180 N, (**d**) 260 N, 50 °C, (**e**) 20 N, (**f**) 80 N, 200 °C. The edge of pins is also shown at the top right corner of the figures. The arrows show the sliding direction.

Under the condition of 0.8 m/s, the dominant wear mechanisms were identified as abrasion + oxidative wear, delamination accompanied by surface oxidation and delamination + mild plastic deformation within the mild wear regime, and severe plastic deformation + spallation of oxide layer, severe plastic deformation and surface melting within severe wear regime. The chemical compositions of worn surfaces examined by EDS analysis are listed in Table 1. At test temperatures of 50 °C and 100 °C, with increasing applied load, wear mechanism transited in such a sequence: abrasion + oxidation→delamination accompanied by surface oxidation→severe plastic deformation + spallation of oxidation layer→surface melting. SEM micrographs of worn surfaces at test temperatures of 50 °C and 100 °C are shown in Figure 2a–f, respectively. Figure 2 also shows the edge of the pins at the top right corners of Figure 2c,d. When the test temperature was 50 °C, at 20 N, the worn surface morphology revealed the grooves parallel to the sliding direction and a few fine powders (Figure 2a). In addition, a high oxygen element content of 14.08% was detected on the worn surface by EDS. These are typical features of abrasion and oxidation wear. When the load was increased to the range of 40–120 N, the worn surface morphology was characterized by a great number of cracks mostly perpendicular to the sliding direction (Figure 2b); meanwhile the oxygen element content still maintained a high level above 10%. The cracks perpendicular to the sliding direction are the most acknowledged feature of delamination wear in magnesium alloys [22,23,24,25,26]. Therefore, the dominant wear mechanism is actually delamination accompanied by surface oxidation. When the applied load was increased above 120 N, the wear behavior was transformed to severe wear regime. In the load range of 140–240 N, the pronounced morphology characteristics of the worn surfaces were the flatten surface without cracks and the extruded-out edge (Figure 2c), indicating a severe plastic deformation of surface layer material. Meanwhile, some spallation of the oxide layer was found at localized locations on the worn surface. At 260 N, the worn surface presented the smoothest morphology due to surface melting effect originating from a large amount of frictional heating between contact surfaces (Figure 2d). At 150 °C, the wear mechanism also transited in a sequence similar to that at 50 °C and 100 °C, except for absence of abrasion wear and spallation of oxide layer. For example, it was found that even subjected to a low load of 20 N, the wear was controlled by delamination accompanied by surface oxidation. The absence of abrasion wear could be due to the improvement of plastic deformation ability by higher test temperature. When test temperature was 200 °C, the surface oxidation effect almost disappeared but a mild plastic deformation occurred in the mild wear regime, since the oxygen element content on the worn surfaces kept in a low level of 1.19–3.84%. In addition, the features of delamination were insignificant at 200 °C, and not as extensive as at 100 °C and 150 °C. The additional slip system of the studied alloy can be effectively activated at such a high temperature, which causes a mild plastic deformation and limited delamination of surface material. These changes may be the reason for a lower wear rate occurred within 20–50 N at 200 °C than at 100 °C and 150 °C. Therefore, the wear mechanisms were identified as delamination + mild plastic deformation at loads of 20–50 N in mild wear regime (Figure 2e), severe plastic deformation at 60–120 N (Figure 2f), and surface melting at loads of 140–160 N in severe wear regime.

**Figure 3:**
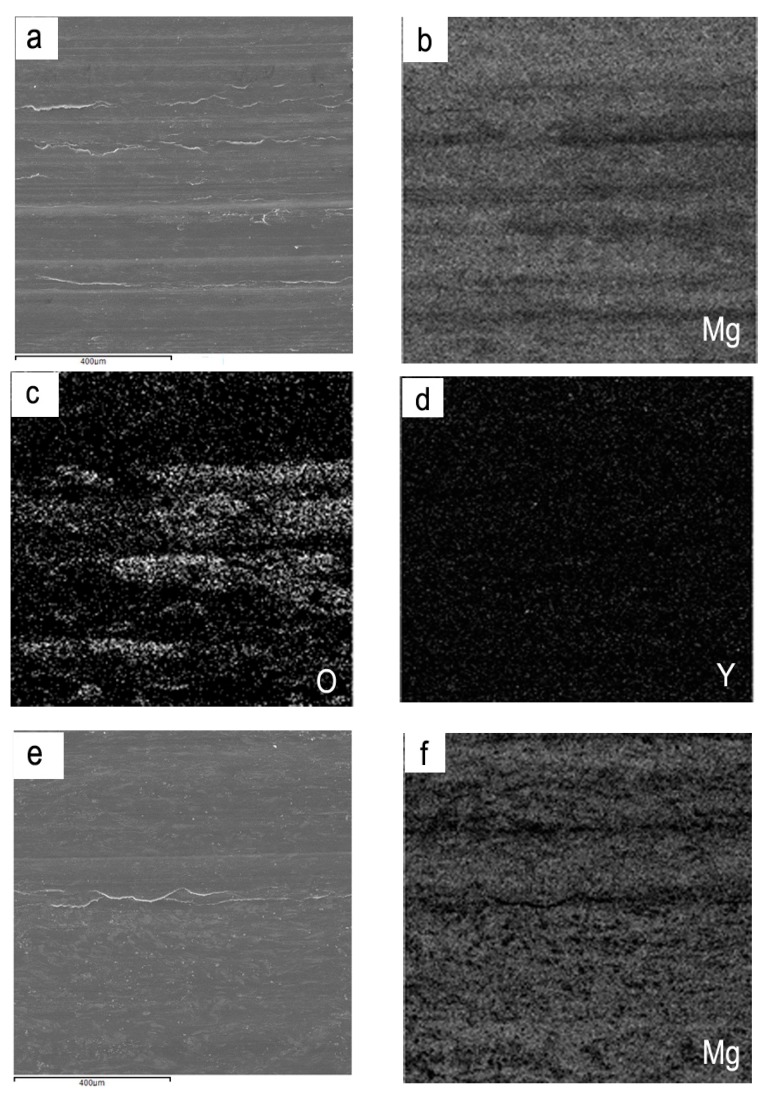

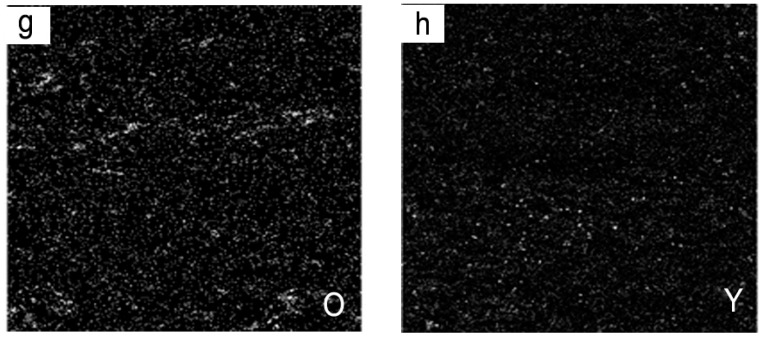
SEM images and EDS mapping of worn surfaces at different applied loads and test temperature under 0.8 m/s: (**a**) SEM image at 120 N, 150 °C, (**b**) O, (**c**), Mg, (**d**) Y, (**e**) SEM image at 80 N, 200 °C, (**f**) O, (**g**) Mg, (**h**) Y.

The phenomenon that there exist plateaus on the wear rate curves regardless of applied load at 150 °C and 200 °C in severe wear regime is contrary to the general wear rate variations in the severe wear regime for conventional Mg alloys such as AZ and AS31 alloys. As for those alloys, the wear rate typically increased rapidly with increasing applied loads in the severe wear regime. Analyses of the elemental mappings of worn surfaces to some extent verify the roles of oxide layer and Mg_12_ZnY phase enrichment in formation of the wear rate plateaus. Figure 3 shows a comparison of elemental mappings on worn surfaces between the two test temperatures. It can be seen that there exists quite a uniform and intact oxide layer on the worn surface subjected to 120 N at 150 °C, the oxygen element content of worn surface reaches as high as 9.24% (Figure 3a–d). However, a number of Y-containing particles are found on the worn surface subjected to 80 N at 200 °C (Figure 3e–g). They are particles of Mg_12_ZnY phase rather than Y-containing oxide, because the yttrium element content of worn surface reaches 8.5%, but oxygen element content is only 2.8%. Therefore, the plateau occurred at 150 °C within 80–140 N could be attributed to a surface oxide layer formation and good coordination between the oxide layer and the alloy substrate, since the oxygen element content kept a high level of 7.67–9.24%, and the spallation extent of the oxide layer was very low within the load range of 80–140 N. Nevertheless, the plateau at 200 °C within 60–80 N apparently has no direct relation with surface oxidation, and could be caused by the short-fiber reinforcement effect of Mg_12_ZnY phase, since the oxygen element content keeps a low level below 3.0% within 60–80 N. The yttrium element content was, however, rather high at the plateau, 8.5% at 80 N, indicating the enrichment of Mg_12_ZnY phase in the surface layer material. The short-fiber reinforcement effect was also found in an extruded Mg97Zn1Y2 alloy where the LPSO phases were aligned along the direction of extrusion [5]. The enrichment of Mg_12_ZnY phase could be attributed to the plastic deformation characteristic and short-fiber reinforcement effect. Under the condition of a proper strain rate of surface layer at 0.8 m/s, with unique deformation feature, the strips of Mg_12_ZnY phase remained intact after bending towards the sliding direction along with the α-Mg dendrites, while the α-Mg dendrites flowed grossly out of the surface but Mg_12_ZnY phase, resulting in an enrichment of Mg_12_ZnY phase in the surface layer. Furthermore, the enrichment of Mg_12_ZnY phase in the surface layer material can even be kept throughout the load range of 60-140 N. Therefore, the short-fiber reinforcement effect of Mg_12_ZnY phase could be the reason for why a lower wear rate occurred at 200 °C rather than at 100 and 150 °C.

**Figure 4:**
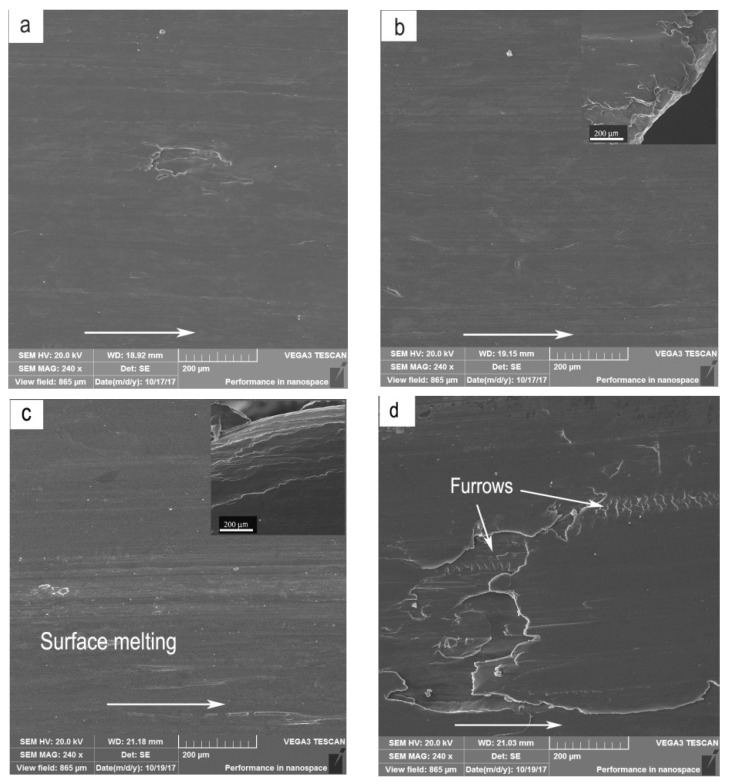

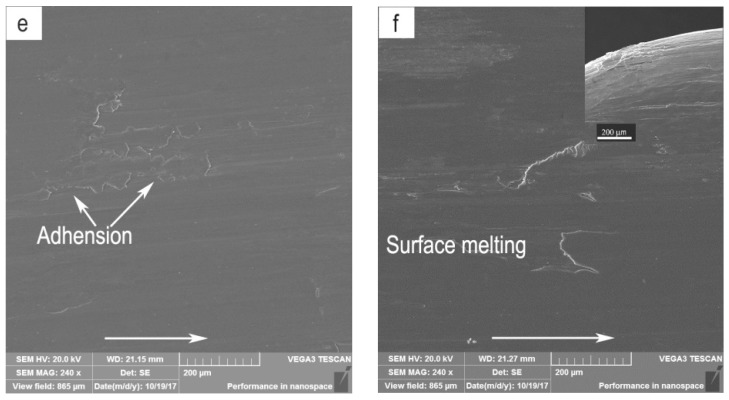
Worn surface morphologies of Mg97Zn1Y2 alloy at different applied loads and test temperatures under 3.0 m/s: (**a**) 20 N, 100 °C, (**b**) 50 N, 100 °C, (**c**) 80 N, 100 °C, (**d**) 20 N, 150 °C, (**e**) 50 N, 150 °C, (**f**) 80 N, 150 °C. The edge of pins is also shown at the top right corner of the figures. The arrows show the sliding direction.

Under the conditions of 3.0 m/s and 4.0 m/s, the dominant wear mechanisms were identified as delamination accompanied by surface oxidation, delamination and delamination + adhesion wear within the mild wear regime, severe plastic deformation, severe plastic deformation + adhesion, and surface melting within the severe wear regime. The EDS results of chemical compositions of worn surfaces are listed in Table 2. The adhesion wear took place mainly at higher test temperatures of 150 °C and 200 °C. At 50 °C and 100 °C, wear mechanism transited with increasing applied load in such a sequence: delamination accompanied by surface oxidation → severe plastic deformation → surface melting. For instance, under the condition of sliding speed 3.0 m/s and test temperature 100 °C, the SEM micrographs of worn surfaces are shown in Figure 4a–f, respectively. Figure 4 also shows the edge of pins at the top right corners of Figure 4b,c,f. At 20 N, large or small irregular scars were observed on the worn surface (Figure 4a), while the oxygen element content was as high as 9.1%, suggesting that the wear mechanism was delamination accompanied by surface oxidation. When the load was increased to the range of 40–80 N, the worn surface experienced a severe plastic deformation, resulting in an extruded edge (Figure 4b). The oxygen element content was only 3.88%. Therefore, the main wear mechanism was severe plastic deformation. When the load reached 100 N, surface melting was the dominant wear mechanism, since an extraordinarily smooth worn surface and waved edge were produced by the melt spreading (Figure 4c). However, at the higher test temperatures of 150 °C and 200 °C, adhesion wear occurred in the load range where delamination wear was controlled, leaving behind a series of scale-like furrows (Figure 4d). In the severe wear regime, the adhesion wear also occurred in load ranges where severe plastic deformation controlled (Figure 4e), and even surface melting controlled (Figure 4f).

**Figure 5:**
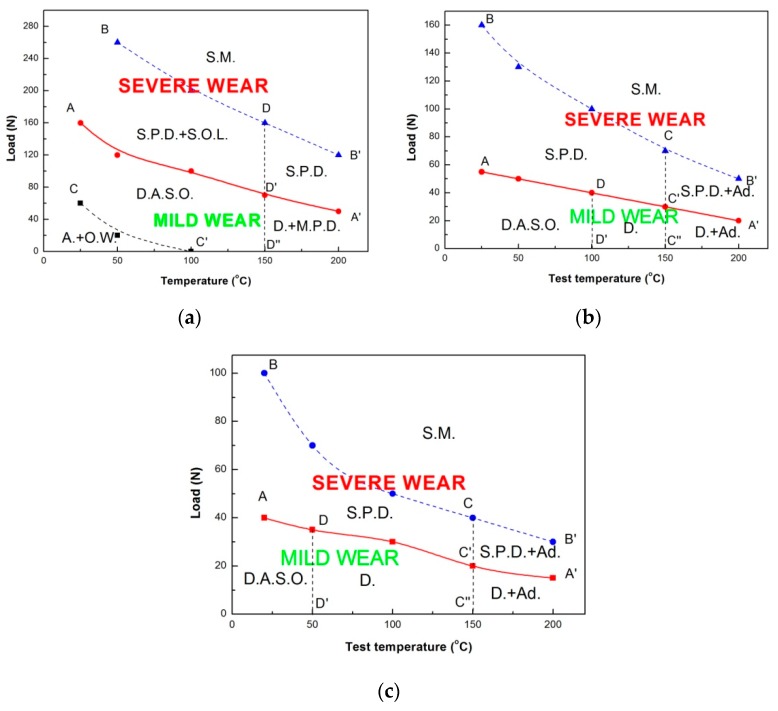
The elevated-temperature wear transition maps: (**a**) 0.8 m/s, (**b**) 3.0 m/s, (**c**) 4.0 m/s. A.—Abrasion; Ad.—Adhesion; O.W.—Oxidative Wear; D.—Delamination; D.A.S.O.—Delamination Accompanied by Surface Oxidation; M.P.D.—Mild Plastic Deformation; S.P.D.—Severe Plastic Deformation; S.O.L.—Spallation of Oxide Layer; S.M.—Surface Melting.

The elevated-temperature wear transition maps of the studied alloy tested at three sliding speeds were established on applied load versus test temperature, as shown in Figure 5. Each map can be divided into two wear regimes by a solid line AA′: Above is mild wear regime, and below is severe wear regime. Under the condition of 0.8 m/s, the mild wear regime consists of three sub-regimes: abrasion + oxidative wear, delamination accompanied by surface oxidation and delamination + mild plastic deformation sub-regimes, while the severe wear regime is composed of three sub-regimes: severe plastic deformation + spallation of oxide layer, severe plastic deformation and surface melting sub-regimes, as shown in Figure 5a. The mild–severe wear transition line AA′ was confirmed by comprehensively considering a series of factors, such as sudden rising of wear rate, extruded edge by naked eyes and occurrence of severe plastic deformation mechanism by SEM. The severe plastic deformation-surface melting transition is indicated by dashed line BB′, while transition from abrasion + oxidative wear to delamination accompanied by surface oxidation by dashed line CC′. The boundary line BB′ was confirmed by SEM observation of surface melting. The boundary line CC′ was established by SEM observation of wear mechanism transition from abrasion to delamination. The boundary line D′D′′ was determined by a high content of O element above 8.21% on the worn surfaces in delamination dominated mechanism. Similarly, the boundary line DD′ between sever plastic deformation + spallation of oxide layer and severe plastic deformation sub-regimes was established by SEM observation of spallation of oxide layer and a high content of O element above 7.67% on the worn surfaces in severe-plastic-deformation dominated mechanism. Under the conditions of 3.0 m/s and 4.0 m/s, the mild wear regime is composed of three sub-regimes: delamination accompanied by surface oxidation, delamination and delamination + adhesion sub-regimes, while severe wear regime consists of three sub-regimes: severe plastic deformation, severe plastic deformation + adhesion and surface melting sub-regimes, as shown in Figure 5b,c respectively. It is found that with increasing sliding speed to 3.0 m/s or 4.0 m/s, in the mild wear regime, the abrasion + oxidative wear sub-regime disappears and a delamination sub-regime occurs between delamination accompanied by surface oxidation and delamination + adhesion sub-regimes. The boundary line DD′ shifts left from 100 °C to 50 °C when sliding speed is increased from 3.0 m/s to 4.0 m/s. Adhesion wear takes place at high temperatures of 150 °C and 200 °C in the mild and severe wear regimes. The most important findings are that the mild–severe wear transition load decreases almost linearly with test temperature within a test temperature range of 20 °C to 200 °C, except for a single data point at 20 °C under the condition of 0.8 m/s, and two data points at 150 °C and 200 °C under the condition of 4.0 m/s. In addition, the overall level of mild–severe wear transition load descends with increasing sliding speed within the temperature range of 20–200 °C, ranging from 160 N to 50 N in the case of 0.8 m/s, 55 N to 20 N in the case of 3.0 m/s, and 40 N to 10 N in the case of 4.0 m/s.

### 3.3. Worn Surface Hardness before and after Mild–Severe Wear Transition

The wear of metallic materials is a complicated surface process that involves in a lot of influence factors including hardness, surface oxidation, mechanically mixed layer (MML) and microstructure transformation [27,28]. Among them, the worn surface hardness could be the most important direct factor [29]. Therefore, in order to explore the mild–severe wear transition mechanism, the hardness of worn surfaces of specimens after sliding at various sliding speeds and test temperatures was firstly measured as a function of applied load.

**Figure 6:**
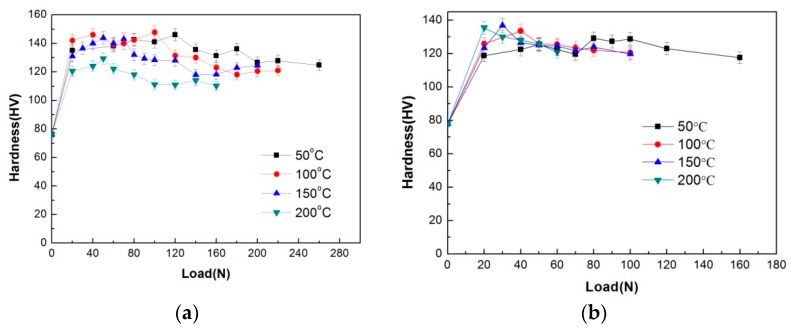
Hardness of worn surface under different sliding speeds: (**a**) 0.8 m/s, (**b**) 3.0 m/s.

Under the condition of 0.8 m/s, it is found that after sliding at different temperatures, all the worn surface hardness curves present a similar variation trend with applied load, i.e. climbing to a peak at a certain load and descending hereafter, as shown in Figure 6a. The surface hardness climbing could be attributed to strain hardening of surface layer material and/or formation of MML. MML could be contributed a little or more to the surface hardness at the climbing stages at test temperatures of 50 °C, 100 °C and 150 °C, because the oxygen element content maintains a high level of 7.67–14.98%. However, at test temperature of 200 °C, the effect of surface oxidation or MML on the worn surface hardness can be ignored since the oxygen element content only ranges from 1.19% to 3.84%. While the hardness descending after peak could not be due to absence of MML, since the difference of oxygen element contents is much small just before and after surface hardness transition load. Moreover, it is noted that the worn surface hardness transition loads at various test temperatures agree well with their respective mild–severe wear transition loads on the whole. Under the condition of 3.0 m/s, the surface hardness variations at different test temperatures are also similar to those under the condition of 0.8 m/s, and the worn surface hardness transition loads are found almost equal to their respective mild–severe wear transition loads, as shown in Figure 6b. The constant repetition of surface softening after mild–severe wear transition therefore should not be just a coincidence; it could be aroused by the property change of surface layer material, which originates from the microstructure transformation during sliding wear test, after all.

### 3.4. Comparison of Microstructures and Hardness in Subsurfaces before and after Mild–Severe Wear Transition

**Figure 7:**
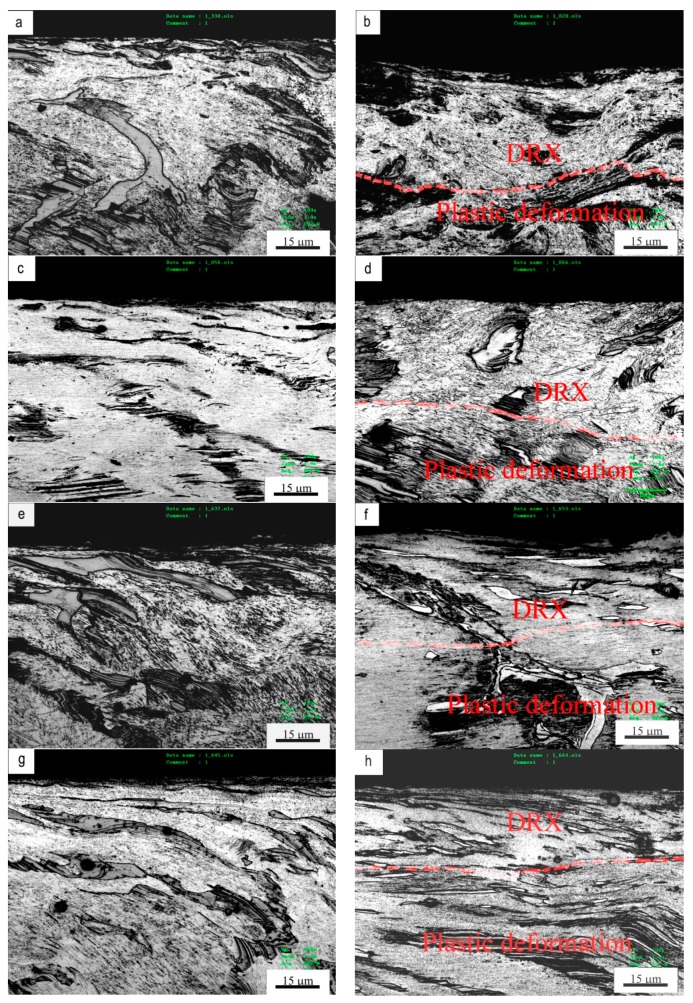
Subsurface microstructures under different sliding conditions: (**a**) 70 N, 100 °C, (**b**) 140 N, 100 °C, (**c**) 40N, 200 °C, (**d**) 100 N, 200 °C, under 0.8 m/s; (**e**) 20 N, 100 °C, (**f**) 60 N, 100 °C, (**g**) 20 N, 200 °C, (**h**) 40 N, 200 °C, under 3.0 m/s.

The cross-sectional micrographs were taken parallel to the sliding direction for investigating microstructure change in subsurfaces before and after mild–severe wear transition, as shown in Figure 7. Under the condition of 0.8 m/s, when test temperature was 100 °C, at 70 N in mild wear regime, friction force and normal load produced a friction-affected zone (FAZ) of about 250 μm thickness beneath surface, where the subsurface material was plastically deformed. The α-Mg dendrites elongated towards sliding direction, while the strips of Mg_12_ZnY phase coordinated with plastic deformation of α-Mg dendrites, also bent towards the sliding direction without breaking, as shown in Figure 7a. At 140 N in severe wear regime, FAZ reached a depth of about 310 μm, and it consisted of two sub-zones: a DRX sub-zone of about 40 μm thickness and a plastic deformation sub-zone of about 270 μm thickness. It was found from the high-magnification photograph that the DRX sub-zone was composed of newly-formed fine grains which were transformed from the plastically deformed microstructure, while most strips of Mg_12_ZnY phase were still kept intact in DRX sub-zone without breaking, as shown in Figure 7b. When the test temperature was increased to 200 °C, at 40 N in mild wear regime, the FAZ was also composed of a plastic deformation zone, extending to a depth of 180 μm, as shown in Figure 7c. At 100 N in severe wear regime, FAZ consisted of a top DRX sub-zone of about 60 μm thickness and a bottom plastic deformation sub-zone of about 200 μm. In the DRX sub-zone, DRX grains were much larger than those formed at 100 °C due to higher test temperature; they nucleated and grew at locations of platelet-shaped LPSO precipitates within the plastically deformed α-Mg dendrites, as shown in Figure 7d. Under the condition of 3.0 m/s, when the test temperature was 100 °C, at 20 N in mild wear regime, FAZ reached a depth of about 220 μm, where the strips of Mg_12_ZnY phase also presented a good flexibility with respect to the deformation with α-Mg dendrites via kink deformation or dislocation motion, as shown in Figure 7e. At 60 N in severe wear regime, FAZ extended to a depth of about 260 μm, a DRX sub-zone of about 30 μm thickness was formed beneath surface, followed by a plastic deformation zone of about 230 μm thickness, as shown in Figure 7f. Subjected to such a high sliding speed, the DRX grains was extremely fine due to a high strain rate produced in the surface layer and a relatively short transformation period. Recently, Zhang et al. [30] explained the nano-grained refinement mechanism by DRX in subsurface of AZ31 magnesium alloy subjected to a large load of 500 N and a sliding speed of 0.2 m/s. In addition, the friction-induced DRX refinement of subsurface microstructure in nanometer scale was also found in pure Cu and Cu-Al alloys [31,32]. Therefore, the depth of DRX sub-zone can only be roughly determined by absence of LPSO lamellas within α-Mg matrix. At 200 °C, the difference between microstructures before and after mild–severe wear transition was also hard to recognize, as shown in Figure 7g,h. Therefore, the absence of LPSO lamellas and light color contrast of etched sub-zone were used to determine the DRX sub-zone.

**Figure 8:**
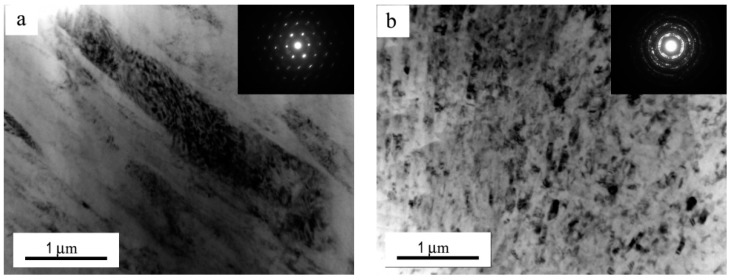
TEM images at 25 μm deep from the top surface for the Mg97Zn1Y2 pins worn under 0.8 m/s: (**a**) 70 N, (**b**) 140 N.

Under the condition of sliding speed 0.8 m/s and test temperature 100 °C, the transformation from the plastically deformed into a fine DRX microstructure in submicrometer and nanometer scale was further verified by TEM observation. Figure 8a,b show TEM micrographs of microstructures at a depth of about 25 μm for pins subjected to 70 N and 140 N, respectively. It is apparent that at 70 N, α-Mg dendrites experience a certain extent of plastic deformation along the sliding direction, and the corresponding selected area diffraction pattern consists of typical sharp spots of the hexagonal close packed (hcp) structure of α-Mg phase, whereas at 140 N, α-Mg dendrites are replaced by the nanometer and sub-micrometer grains, and the corresponding selected area diffraction pattern is composed of elongated spots and continuous diffraction rings, indicating that randomly oriented grains were obtained by DRX.

**Figure 9:**
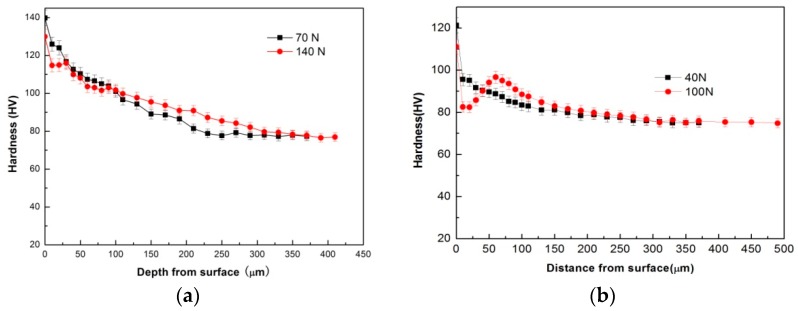

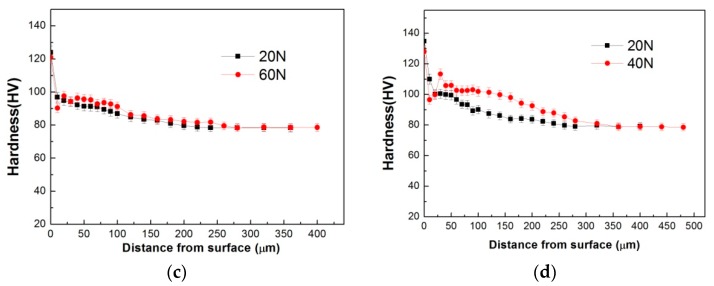
Hardness vs. depth from surface for Mg97Zn1Y2 alloy pins worn at 100 °C and 200 °C under different sliding speeds: (**a**) 100 °C, (**b**) 200 °C, 0.8 m/s, (**c**) 100 °C, (**d**) 200 °C, 3.0 m/s.

DRX transformation can soften the deformed material. Therefore, in order to identify the friction-induced softening of DRX sub-zone after mild–severe wear transition, microhardness measurements were taken along the depth on several specimens subjected to different loads and test temperatures under the conditions of 0.8 m/s and 3.0 m/s, as illustrated in Figure 9. It can be seen from Figure 9a,b that under the condition of 0.8 m/s, both the hardness curves that were obtained at 70 N and 100 °C, and 40 N at 200 °C in mild wear regime, present a monotonous decreasing with increasing depth until to the hardness of substrate material, about 80 HV. The monotonous decreasing is apparently ascribed to the weakening effect of strain hardening with increasing depth. However, the hardness curves obtained at 140 N and 100 °C, and 100 N at 200 °C in sever wear regime, demonstrate much lower hardness values in the near surface regions than those in the mild wear regime, and a V-shaped valley is formed. The widths of low hardness valleys are 40 μm at 100 °C and 60 μm at 200 °C respectively, which are almost equal to the microstructural observation results shown in Figure 7. This suggests that material softening effect occurs in the near surface region. Considering the observed microstructure transformation from the plastically deformed to the dynamically recrystallized before and after mild–severe wear transition, it can be verified that the softening originates from the DRX realization of surface material. Similarly, the hardness curves obtained under the condition of 3.0 m/s also identified the softening effect in the near surface region after mild–severe transition, as shown in Figure 9c,d.

### 3.5. Evaluating Transition Load by the Contact Surface DRX Temperature Criterion

The changes of microstructure and hardness in subsurfaces prove that DRX realization in subsurface at elevated temperatures is still the reason for mild–severe wear transition. Therefore, our previously proposed contact surface DRX temperature criterion for mild–severe wear transition in room-temperature wear environment can be applied to the elevated-temperature wear, that is, when contact surface temperature *T_s_* reaches the critical DRX temperature *T_DRX_*, *T_s_* ≥ *T_DRX_*, the mild wear transits into severe wear within the test temperature range of 50–200 °C. Lim and Ashby [33] proposed a contact surface temperature relationship with applied load *F*, sliding speed *v* and test temperature *T_o_*, which can be expressed by Equation (1).

(1) $$T_{S}=T_{0}+\frac{\alpha \mu Fvl_{b}}{A_{n}K_{mp}}$$

where *α* is the fraction of the heat conducted into the pin, *μ* the coefficient of friction, *A_n_* the nominal contact area, *l_b_* the mean diffusion distance, *K_mp_* the thermal conductivity of the pin. Under each given sliding speed, the parameters *α*, *l_b_*, *A_n_*, *K_mp_* in Equation (1) can be regarded as approximate constants within the temperature range of 50–200 °C. For example, the deference in *K_mp_* of Mg97Zn1Y2 alloys is small within test temperature range of 50–200 °C, ranging from 57 to 64 Wm^−1^K^−1^ [34]. Therefore, when mild wear transits into severe wear, the transition load *F_T_* can be expressed as Equation (2) using the corresponding critical surface DRX temperature *T_DRX_*, test temperature *T_o_*, sliding speed *v*, coefficient of friction *μ* and a simplified constant *K_DRX_*. *K_DRX_* can be approximately regarded as a constant, which is related with test equipment and material properties of pin and disk, as shown in Equation (3).

(2) $$F_{T}=\frac{\left(T_{DRX}-T_{0}\right)}{K_{DRX}\mu v}$$

(3) $$K_{DRX}=\frac{\alpha l_{b}}{A_{n}K_{mp}}$$

**Figure 10:**
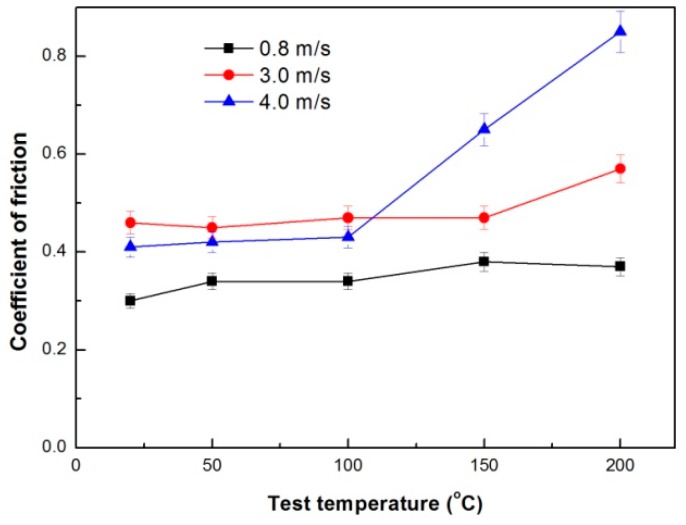
Variations in coefficients of friction at mild–severe wear transition loads with test temperature under different sliding speeds.

The *T_DRX_* values are 317.3 °C at 0.8 m/s, 336.9 °C at 3.0 m/s and 340.7 °C at 4.0 m/s, respectively [13]. Therefore, under each given sliding speed condition, the mild–severe wear transition load *F_T_* should have an almost linear relationship with test temperature *T*_0_, if the coefficient of friction varies little within 50–200 °C. The *K_DRX_* values are determined to be 7.56, 4.25 and 4.94 using the parameters obtained at 50 °C: *T_DRX_* 317.3 °C, *F_T_* 130 N and *μ* 0.34 under the condition of 0.8 m/s, *T_DRX_* 336.9 °C, *F_T_* 50 N and *μ* 0.45 under the condition of 3.0 m/s, and *T_DRX_* 340.7 °C, *F_T_* 35 N and *μ* 0.42 under the condition of 4.0 m/s. It is found that the coefficients of friction at transition loads are influenced by both the sliding speed and test temperature, as shown in Figure 10. With increasing sliding speed from 0.8 m/s to 4.0 m/s, the coefficient of friction increases from a narrow range of 0.30−0.38 to a wide range of 0.41–0.85. Furthermore, it notes that when the sliding speed are 3.0 m/s and 4.0 m/s, the coefficients of friction are much larger at high temperatures of 150 °C and 200 °C than those at low temperatures of 20–100 °C, for example, 0.57 at 200 °C under the condition of 3.0 m/s, 0.65 at 150 °C and 0.85 at 200 °C under the condition of 4.0 m/s. The rapid rising of coefficients of friction is apparently ascribed to the adhesion wear occurred at higher test temperatures of 150 °C and 200 °C under conditions of 3.0 m/s and 4.0 m/s.

**Figure 11:**
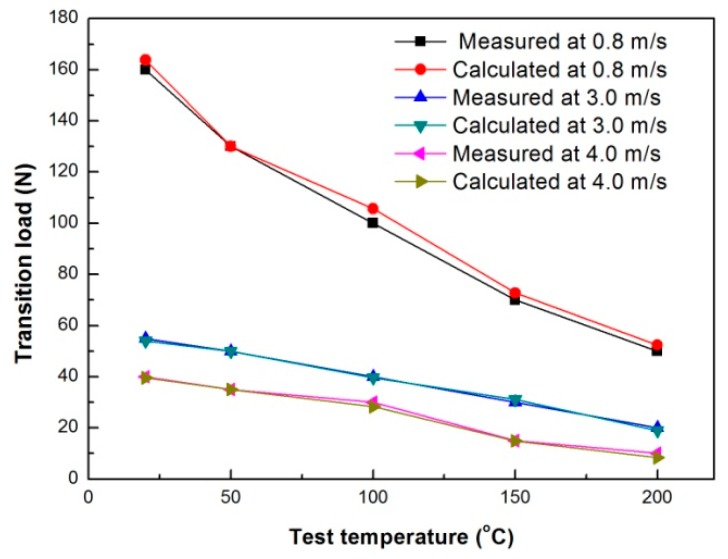
Measured and calculated elevated-temperature mild–severe wear transition loads vs. test temperature.

Under each sliding speed, the transition loads at test temperatures of 20–200 °C can be calculated by introducing their respective coefficients of friction into Equation (2). The differences between the measured and calculated transition loads under different sliding conditions are shown in Figure 11. The calculated transition loads are almost consistent with the measured ones, except for a large deviation 5.0 N at 100 °C under the condition of 0.8 m/s. Therefore, the contact surface DRX temperature criterion that was initially proposed for evaluating mild–severe wear transition at room temperature can also be applied to the mild–severe wear transition at test temperatures of 50–200 °C.

## 4. Conclusions

1. The wear rate of Mg97Zn1Y2 alloy was strongly influenced by applied load and test temperature, especially under high sliding speed conditions of 3.0 m/s and 4.0 m/s. Under the conditions of 3.0 m/s and 4.0 m/s, at test temperatures of 20–200 °C, the slope of wear rate-applied load curve increased considerably with increasing test temperature.

2. In the mild wear regime, abrasion + oxidative wear, delamination accompanied by surface oxidation and delamination + mild plastic deformation were the dominant wear mechanisms under the condition of 0.8 m/s, while delamination accompanied by surface oxidation, delamination and delamination + adhesion were the main wear mechanisms under the conditions of 3.0 m/s and 4.0 m/s. In the severe wear regime, severe plastic deformation + spallation of oxide layer, severe plastic deformation and surface melting were the dominant wear mechanisms under the condition of 0.8 m/s, while severe plastic deformation, severe plastic deformation + adhesion and surface melting were the main wear mechanisms under the conditions of 3.0 m/s and 4.0 m/s.

3. Under the given sliding speed conditions, the mild–severe wear transition load decreases almost linearly within a test temperature range of 50 °C to 200 °C, and the overall level of mild–severe wear transition load vs. test temperature decreases with increasing sliding speed.

4. The mechanism of the mild–severe wear transition at test temperatures of 50–200 °C is the subsurface softening originating from friction-induced DRX realization.

5. The mild–severe wear transition at 50–200 °C follows the contact surface DRX temperature criterion.

## Figures and Tables

**Table 1:** Chemical composition of worn surfaces at 0.8 m/s (wt.%).

Temperature (°C)	Wear Regime	Load (N)	O	Y	Zn
50	Mild	20	14.08	5.87	2.09
		60	10.80	6.19	2.11
	Severe	140	10.66	6.43	2.04
		180	12.72	6.09	2.02
		220	6.76	6.74	2.11
100	Mild	20	14.98	5.84	2.07
		60	9.98	6.28	2.20
	Severe	120	9.90	6.59	2.17
		180	10.74	6.17	2.13
150	Mild	20	8.21	6.62	2.19
		40	8.34	6.58	2.32
	Severe	80	7.67	6.76	2.16
		120	9.24	6.53	2.07
		160	2.84	7.84	2.53
200	Mild	20	3.84	7.39	2.41
		40	3.40	7.39	2.33
	Severe	80	2.28	8.52	2.48
		140	1.19	8.75	2.65

**Table 2:** Chemical composition of worn surfaces at 3.0 m/s (wt.%).

Temperature (°C)	Wear Regime	Load (N)	O	Y	Zn
50	Mild	20	9.01	6.28	2.28
	Severe	60	5.65	6.62	2.36
		90	2.22	6.88	2.22
		100	2.95	7.11	2.36
		120	3.32	6.84	2.22
		160	3.34	6.76	2.33
100	Mild	20	7.23	6.74	2.32
	Severe	50	3.88	7.18	2.29
		60	5.57	6.33	2.20
		80	2.38	6.77	2.36
		100	4.46	7.18	2.29
150	Mild	20	4.12	6.39	2.17
	Severe	40	3.80	6.90	2.23
		50	4.39	6.67	2.26
		70	1.28	6.79	2.25
		80	4.68	6.69	2.13
200	Mild	20	3.84	6.63	2.31
	Severe	30	3.04	6.67	2.12
		40	1.15	6.84	2.27
		50	2.30	6.73	2.32
		60	1.18	6.73	2.33
